# New Medical Journals

**Published:** 1858-09

**Authors:** 


NEW MEDICAL JOURNALS.—We place, with great pleasure, the following journals on our exchange list:—*Maine Medical and Surgical Reporter*, edited by Drs. Richardson and Cummings; *Oglethorpe Medical and Surgical Journal*, edited by Drs. Boyd and Steele; *Savannah Journal of Medicine*, edited by Drs. Sullivan, Harris, and Arnold; *Medical Journal of North Carolina*, edited by Dr. Edward Warren.

